# Supplementary material for: Identification of photoperiod-induced specific miRNAs in the adrenal glands of Sunite sheep (Ovis aries)
Source: Front Vet Sci. 2022 Jul 22;9:888207. doi: 10.3389/fvets.2022.888207 (PMC9354845; doi:10.3389/fvets.2022.888207)
Supplement: Supplementary file 1 [file Data_Sheet_1.ZIP › Supplementary file/miRDeep2 file.zip!/miRDeep2 file/Know_Structure/SPLP42A2/oar-mir-194.pdf]

miRBase precursor : oar-mir-194
Total read count : 4139
oar-miR-194 read count : 4130
remaining reads : 9

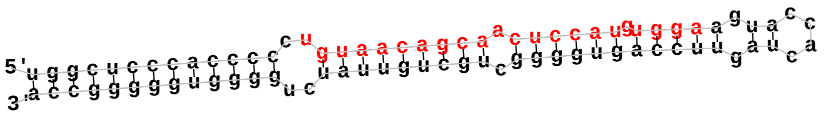

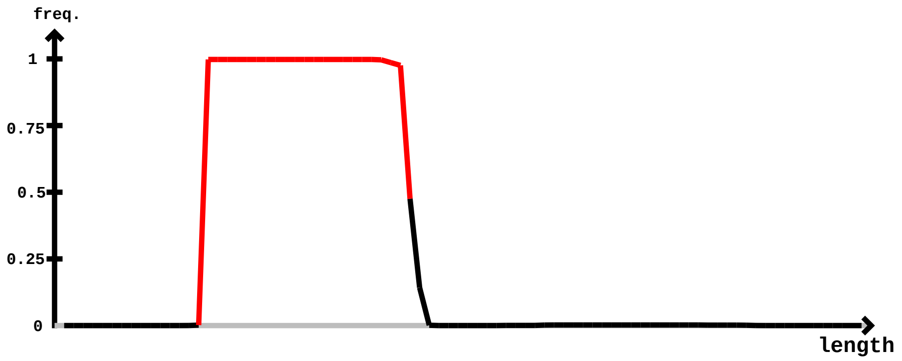


```
                oar-miR-194
5'- uggcucccacccccuguaacagcaacuccaugugggaaguaccacuaguuccaguggggcugcuguuaucugggguggggcca  -3'   exp
    ((((((((((((..(((((((((.((((((.(((((.((....)).))))))))))).)))))))))..))))))))))))     reads     mm     sample
    ............ccuguaacagcaacuccaugug...............................................    1         0      seq
    .............cuguaacagcaacuccaugugg..............................................    3         0      seq
    .............cuguaacagcaacuccaugugga.............................................    2         0      seq
    .............cuguaacagcaacuccauguggaa............................................    1         0      seq
    ..............uguaacagcaacuccaug.................................................    6         0      seq
    ..............uguaacagcaacuccaugu................................................    43        0      seq
    ..............uguaacagcaacuccaugug...............................................    42        0      seq
    ..............uguaacagcaacuccaugugg..............................................    2068      0      seq
    ..............uguaacagcaacuccaugugga.............................................    1377      0      seq
    ..............uguaacagcaacuccauguggaC............................................    50        1      seq
    ..............uguaacagcaacuccauguggaa............................................    532       0      seq
    ..............uguaacagcaacuccauguggaag...........................................    5         0      seq
    ..........................................aguuccaguggggcugcuguu.................    2         0      seq
    ..............................................ccaguggggcugcuguuaucu.............    2         0      seq
    ..............................................ccaguggggcugcuguuaucug............    4         0      seq
    ...............................................caguggggcugcuguuaucugg...........    1         0      seq
```
